# Review of: The Cancer Crisis in Appalachia Kentucky Students Take Action

**DOI:** 10.13023/jah.0302.07

**Published:** 2021-05-03

**Authors:** Stephenie Kennedy-Rea

**Affiliations:** West Virginia University Cancer Institute

**Keywords:** Appalachia, cancer, book review, students

## Abstract

The *Journal of Appalachian Health* is committed to reviewing published media that relates to contemporary concepts affecting the health of Appalachia. With cancer mortality rates higher in rural and Appalachian communities, a focus on how cancer impacts our families and communities is more important than ever. Dr. Stephenie Kennedy-Rea reviews the book *The Cancer Crisis in Appalachia: Kentucky Students Take Action*.

## MEDIA TYPE: BOOK

### CITATION

Vanderford NL, Hudson L, Prichard C, et al. *The Cancer Crisis in Appalachia: Kentucky Students Take Action*. Lexington KY: The University Press of Kentucky. 2020. ISBN 978-1-950690-03-9; Cost: $19.95 paperback, $18.95 Kindle edition.

### ABOUT THE REVIEWER

Stephenie Kennedy-Rea is an Associate Center Director at the WVU Cancer Institute and a faculty member in the division of hematology oncology at the WVU School of Medicine. She directs the office of Cancer Prevention and Control, the outreach, education, and population-based research arm of the Cancer Institute. She oversees eight programs aimed at decreasing the cancer burden in the state. Additionally, she leads the Community Engagement and Outreach Core for the WV Clinical and Translational Science Institute and serves as the Director of the Mountaineer Health Initiative.

### ABOUT THE AUTHOR

This hope-filled book is a compilation of essays about growing up in eastern Kentucky, the challenges associated with life in Appalachia, and personal narratives of how cancer affects families and communities. Most of the essays are written by high school sophomores and juniors with a few penned by college undergraduates; all the authors participated in a University of Kentucky summer training program. Editors include Nathan L. Vanderford, Lauren Hudson, and Chris Prichard.

### THE REVIEW

This book is for anyone interested in how cancer affects a family. The essays illuminate the ways cancer changes the lives of children who witness the struggles of parents, grandparents, and family friends. Clinicians, parents, researchers, and patients can learn how this pain can be harnessed into thoughtful community-based solutions. In the summer of 2019, as a result of participation in the University of Kentucky Markey Cancer Center’s Appalachian Career Training in Oncology (ACTION) Program, a group of forward-thinking students took on the challenge of writing about their personal experiences with cancer as well as what they learned regarding cancer statistics, prevention, early detection, and social determinants of health. Most concluded each essay with potential solutions to the cancer problems that plague Appalachia.

The result is a thoughtful collection of essays by high school and undergraduate students that challenge the reader to look at cancer from many different angles. The writers, most of whom are high school juniors and seniors, put a face on the statistics and make real the challenges encountered by Appalachian cancer patients and their families. They speak of access to care hardships, poverty, fatalism, and the tobacco and obesity crises, but they also recognize the strengths of family, church, and community. These young people articulate the role of education in mitigating hardships and believe that hope rests in research and prevention. The ACTION participants take the data and science learned at the University of Kentucky and view their experiences and communities through those lenses.

They then put forward a wide array of community-based solutions that start with implementing prevention programs in schools, educating adults, and improving access to cancer treatment at the local level. Some dream big and write of improved treatments, more NCI-designated cancer centers, and increased economic opportunities in eastern Kentucky. Sprinkled throughout the stories and statistics are pictures, quotes, and even some poetry. These creative, intelligent, and energetic young people use words to paint a call to action that addresses the challenges of cancer in Appalachia. The reader walks away with a sense of purpose, a desire to roll up one’s sleeves to tackle the challenges, and an understanding that the next generation is poised to tackle cancer care across the continuum from prevention through survivorship.

Publisher link: https://www.kentuckypress.com/9781950690039/the-cancer-crisis-in-appalachia/

The topic of cancer in Appalachia is covered in several recent publications in the Journal of Appalachian Health:

Hudson L, Sharp K, Prichard C, Ickes M, Alameh S, Vanderford NL. Cancer Curriculum for Appalachian Kentucky Middle and High Schools. J Appalach Health 2021;3(1):43–55. DOI: https://doi.org/10.13023/jah.0301.05
Boatman D, Eason S, Conn ME, Miller S, Kennedy-Rea S. Advancing cancer prevention practice facilitation work in rural primary care during COVID-19. J Appalach Health 2020;2(4):4–10. DOI: https://doi.org/10.13023/jah.0204.02
Scutchfield FD, Patrick K. Introducing the L.A.U.N.C.H. Collaborative. J Appalach Health 2020;2(1):1–5. DOI: https://doi.org/10.13023/jah.0201.01.
Hesse BW, Ahern DK, Ellison M, et al. Barn-raising on the digital frontier: The L.A.U.N.C.H. Collaborative. J Appalach Health 2020;2(1):6–20. DOI: https://doi.org/10.13023/jah.0201.02
Ellison PM, Vanderpool RC. Preface: Experiencing Cancer in Appalachian Kentucky. J Appalach Health 2020;2(3):69–73. DOI: https://doi.org/10.13023/jah.0203.08
McComsey M, Ahern DK, Vanderpool RC, et al. Experiencing Cancer in Appalachian Kentucky. J Appalach Health 2020;2(3):74–116. DOI: https://doi.org/10.13023/jah.0203.09
Baus AD, Wright LE, Kennedy-Rea S, et al. Leveraging electronic health records data for enhanced colorectal cancer screening efforts. J Appalach Health 2020;2(4):53–63. DOI: https://doi.org/10.13023/jah.0204.07

#### From the NIH Director’s Blog

Dr. Francis S. Collins:

It was wonderful to have First Lady Jill Biden pay a virtual visit to NIH on February 3, 2021, on the eve of World Cancer Day. Dr. Biden joined me, National Cancer Institute (NCI) Director Ned Sharpless, and several NCI scientists to discuss recent advances in fighting cancer. On behalf of the entire NIH community, I thanked the First Lady for her decades of advocacy on behalf of cancer education, prevention, and research.

## Figures and Tables

**Figure 1:**
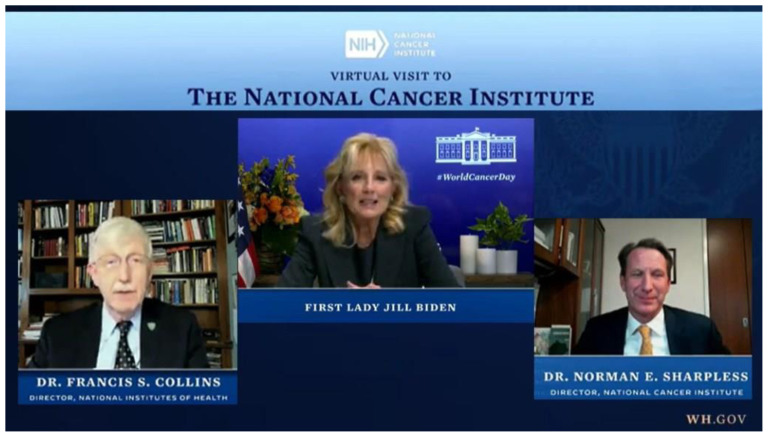
Credit: Adapted from White House video. **Note that the book on the desk behind Dr. Sharpless is *The Cancer Crisis in Appalachia: Kentucky Students Take Action*.

